# The Risk Perception of the Chinese Diaspora during the COVID-19 Pandemic: Targeting Cognitive Dissonance through Storytelling

**DOI:** 10.3390/ijerph21050556

**Published:** 2024-04-27

**Authors:** Doris Yuet Lan Leung, Shoilee Khan, Hilary Hwu, Aaida Mamuji, Jack Rozdilsky, Terri Chu, Charlotte Lee

**Affiliations:** 1 School of Nursing, The Hong Kong Polytechnic University, Hong Kong, China; 2 Faculty of Liberal Arts & Professional Studies, York University, Toronto, ON M3J 1P3, Canada; shoilee.khan@gmail.com (S.K.); amamuji@yorku.ca (A.M.); rozdilsk@yorku.ca (J.R.); terrichu@yorku.ca (T.C.); 3 Daphne Cockwell School of Nursing, Toronto Metropolitan University, Toronto, ON M5B 2K3, Canada; hhwu@torontomu.ca (H.H.); lee.charlotte@torontomu.ca (C.L.)

**Keywords:** cognitive dissonance, COVID-19, immigrants, risk communication, risk perception, qualitative, narrative, storytelling, vulnerability

## Abstract

The global COVID-19 pandemic in 2020 required risk communications to mitigate the virus’ spread. However, social media not only conveyed health information to minimize the contagion, but also distracted from the threat by linking it to an externalized ‘other’—primarily those appearing to be of Chinese descent. This disinformation caused the attribution of blame to Chinese people worldwide. In Canada’s Greater Toronto Area, Chinese individuals reported widespread public stigma that compounded their risk of contagion; to the degree that it was driven by cognitive dissonance, it generated experiences of social and cultural vulnerability. In this paper, we draw on the aforementioned study’s findings to explain how the risk perception and threat appraisal of Chinese diaspora individuals were impacted by different cognitive dissonance pathways. These findings explore how storytelling is a viable intervention with which to target and mitigate cognitive dissonance. Indeed, the mechanisms of cognitive dissonance can modify risk perception and mitigate social and cultural vulnerability, thereby averting potential long-term negative consequences for one’s mental health and well-being. We hope our guidance, training educators to target pathways of cognitive dissonance by drawing on storytelling (with humour), can assist them to better convey information in ways that are more inclusive during public health emergencies.

## 1. Introduction

On January 30, 2020, the World Health Organization (WHO) announced that the novel coronavirus (COVID-19) was a public health emergency requiring a coordinated international response [1]. This prompted authorities to intensify risk communication via social media to mitigate the spread [1]. However, while social media was useful for conveying key public health information, it also spread instances of anti-Chinese stigma globally [2]. Instances of this also occurred in Canada after the first case of COVID-19 was confirmed in late January 2020 [3].

On January 22, 2020, a 56-year-old man went by ambulance to Toronto’s Sunnybrook Hospital due to flu-like symptoms. One day earlier, he had returned from a three-month visit to the Wuhan region of China. On January 25, laboratory tests confirmed he was infected by a coronavirus, which we now know to have been COVID-19 [3]. This fact likely contributed to the ethnic stigma experienced, which is discussed later in this paper, as unfounded political and internalized public biases were directed towards East Asian persons from January to July 2020, during the first wave of the COVID-19 pandemic. Indeed, in a poll of Canadians from April 2020, commissioned by Corbett Communications, respondents in Toronto, Montreal, and Vancouver reported that they either ‘believed that all Chinese or Asian people carry the Coronavirus (4%) or were uncertain about that (10%)’ [4] (p. 1). Across the country, this sentiment was called a ‘shadow pandemic’ by the Angus Reid Institute in June 2020, when a survey of 500 Canadians of Chinese ethnicity reported that exposure to discriminatory behaviours had affected their sense of self and belonging in Canada [5].

### 1.1. Risk Perception

Risk perception is one’s awareness of and receptivity to vulnerability from a threat, (whether or not it is actualized), and the need to appraise, mitigate, and cope with its potential adverse consequences [6]. According to Demirtaş-Madran [7], ‘individuals’ attributions of the cause of disease determine their responses toward the real or perceived disease carriers’ (p. 9); that is, one’s risk perception depends on whether one is considered to be more or less at risk. According to the Extended Parallel Process Model (EPPM), risk perception is not based on an ‘actual’ threat as much as one’s perception of a threat that motivates action [8]. Moreover, risk perception is based on the exogenous components of health communications that shape one’s self-efficacy and response-efficacy [8]. These include the perceived severity of (i.e., significance or magnitude) and perceived susceptibility to the threat [8]. 

When one identifies with a community on the grounds of cultural identity or heritage, one’s risk perception may shift discernibly to the degree that one experiences vulnerability to public stigma [9]. Hullett and Wittes’ [10] study attributed this shift to the dominance of anxiety processes that might compete with the cognitive uncertainty of one’s situation. In doing so, fear can be counter-productive and reduce one’s self- and response-efficacy to adapt [8]. Hence, one’s risk perception is dependent upon one’s agency (self-efficacy), with their social supports, and the social determinants (e.g., socio–economic status, neighbourhood) that shape one’s attitudes and beliefs (response-efficacy). 

In this study, we chose to focus on the Chinese diaspora, defined as a group that self-identifies ethnically as Chinese via birth or ancestry, and lives outside their country of ethnic origin [11]. This group was chosen because they were more likely to experience a greater risk of social vulnerability than other Asian groups, due to their ethnicity, which was directly associated with the origins of the COVID-19 virus [12]. The threat to one’s social vulnerability was conceptualized as a threat to their social norms, created by a ‘shared ideological recognition of national/cultural identity’ [13] (p. 214). 

This paper follows from the results from part one of a qualitative study that used secondary data analysis to explore the risk perception of the Chinese diaspora in the Greater Toronto Area (GTA) in Ontario, Canada, during the first wave of COVID-19 [14]. The results from part one demonstrate that all the Chinese diaspora participants in the latter study experienced vulnerability to public stigma. Moreover, their experiences were generated by cognitive dissonance—of contagion versus stigma—that propelled their threat appraisal of their social and cultural vulnerability. In other words, the participants’ cognitive dissonance pathways shaped their varied perceptions and responses to their vulnerability (i.e., risk perceptions [4]). In this paper, we draw on the aforementioned study’s findings [14] to explain how the risk perceptions of Chinese diaspora individuals were impacted by different cognitive dissonance pathways. To begin, we define cognitive dissonance and the potential variance in cognitive dissonance pathways. 

### 1.2. Cognitive Dissonance

According to Cooper [15], cognitive dissonance (a term coined by Festinger) is a mental discomfort or tension resulting from holding two conflicting beliefs, values, or attitudes that drive the individual to seek information and resources consistent with one’s attitudes and beliefs to alleviate this tension. The drive to resolve cognitive dissonance is motivated by uncertainty or the sense that one is at odds with social mores [15]. This may apply when personal decisions or the decisions of others appear discrepant with or contrary to past beliefs in similar situations; the conflict fuels the individual’s motivation to resolve this dissonance [15]. For example, one may believe all persons should be treated fairly, as in the ‘just world belief’, yet the same persons derogate and blame innocent others for something they are not responsible for, such as the spread of COVID-19 [16]. This generates feelings of tension, in the form of guilt, and motivates one to resolve it. Resolution occurs when one arrives at the sense that they are emotionally correct and their opinions are deemed appropriate and consistent in comparison to those of similar others [15]. To this end, one may adopt several strategies, including (but not limited to) social comparison to alter one’s attitude and mitigate one’s conflict [15]. 

Cooper [15] asserts that the cognitive dissonance pathway generates attitudinal and/or behavioural change *only if* (i) the decision is freely chosen, (ii) the decision is consciously acted upon and reflects responsibility for the decision, (iii) the decision produces or is anticipated to produce an undesirable consequence, and (iv) the (unwanted) consequence is foreseeable at the time of the choice or decision. Hence, the cognitive dissonance pathway is triggered by the anticipation or subsequent realization that the adverse events occurred as a result of one’s actions and, thus, one may be motivated to change ‘to render the (*responsibility and*) consequences of one’s behaviour non-aversive’ [15] (p. 5, italics added). If these conditions are *not* met—that is, if decisions are *not* freely chosen, and/or one is *not* felt to be responsible for the decision at the time of the decision (i.e., undesirable consequences were unforeseen)—attitudinal or behavioural changes *will not occur* [15]. Taking the aforementioned example, change will not occur if a reasonable response to resolve the tension is not felt to be feasible or one is not felt to be culpable [16]. 

### 1.3. Context of Stigma towards Chinese Diaspora in Canada

In Canada, 61% of 1000 Canadians polled during March 2020 reported that they did not think it was acceptable to refer to COVID-19 as the ‘Chinese virus’ or ‘Chinese flu’, [17]; yet, Canadians of Chinese descent still faced high levels of COVID-19-induced racism. In the Angus Reid Institute survey of 500 Canadians of Chinese ethnicity [5], 50% stated that they reported being called names or insults as the direct result of the COVID-19 outbreak. Of these, 43% stated that they experienced being threatened or intimidated. This discrepancy suggests the lack of culpability and/or feasibility of helping Chinese persons who were unfairly victimized during the COVID-19 pandemic in Canada. In this paper, the authors follow up on this hypocrisy and how it impacted the Chinese diaspora in Canada. 

We define stigma, as one of the seminal studies of Goffman describes, as ‘tribal stigma’ in reference to a negative label assigned to a group, in order to shame, discredit, or devalue them as a consequence of their individual race, nationality, and/or religion [18]. According to Aguilar et al. [16], racial prejudice can be a result of deep-rooted and historical implicit bias from social norms (reflected in language and taken-for-granted practices), such that one may not recognize when he or she does not uphold an advocated belief, or may even lay blame on innocent victims due to the ‘just world belief’ (i.e., bad things happen to bad people). This is a form of hypocrisy—that is, acting against advocated beliefs—and is a defensive response to unrecognized cognitive dissonance [16]. 

This study was divided into two parts. The first paper [14] described the methods and results of the theoretical model in detail; these are briefly summarized below. See ‘Understanding the risk of social vulnerability for the Chinese diaspora during the COVID-19 pandemic: A model driving risk perception and threat appraisal of risk communication—A qualitative study’ [14] for the theoretical model used in this paper (part two). The purpose of this paper is to explain the process of how participants’ social vulnerability was impacted by different types of cognitive dissonance, beginning with their identification with their cultural identity. We then discuss the implications of the results for health risk communicators, in order to address the social vulnerability of the Chinese diaspora or similar groups during the current or future pandemics. 

## 2. Material and Methods

### 2.1. Study Design, Location, and Research Question

This study used qualitative data from a descriptive study [2] exploring emergency management actions to address anti-Chinese stigma and the social vulnerability of the Chinese diaspora in the Greater Toronto Area (GTA); this is the largest metropolitan area with a sizeable Chinese diaspora community, that is, 11% in Toronto and 45% in Markham, the city just north of Toronto [2]. This study is a secondary data analysis based on data collected by Mamuji et al. [2] to explain the social vulnerability of the Chinese diaspora and, in doing so, develops a theoretical model of their vulnerability. Specifically, the present study asks the research question: what mechanisms and structures underpinned risk perception and threat appraisal associated with pandemic challenges experienced by Canada’s Chinese diaspora living in the GTA during the first wave of COVID-19? 

The data collection took place between March and May 2020 in Canada’s GTA, which has a population of approximately 6.2 million [19]. The Chinese groups differ with respect to dialect, culture, and political beliefs [20]. As a vulnerable immigrant group during COVID-19, this sample was sufficiently heterogeneous to allow researchers to reveal multiple social barriers (i.e., beyond ethnicity and English language fluency) to seeking help during COVID-19 [21]. 

### 2.2. Theoretical–Conceptual Framework

We adopted a critical realist paradigm, which assumes that multiple mechanisms and social structures impact one’s agency [22]. Individual agency depends on the components of a stratified social reality on three levels: the ‘empirical’—what we tell each other or observe, the ‘actual’—what events occur, whether tangible or not, and the ‘real’—when multiple mechanisms interact with social structures to produce modicum patterns or tendencies for outcomes to occur [22]. 

### 2.3. Researcher Characteristics and Reflexivity

Given this study’s qualitative nature, the researchers were the instrument and their reflexivity to their own assumptions shaped the analysis. The members of our research team have expertise in disaster and emergency management research (AM, JR), and five of the team members identified with a visible minority (DL, CL, HH, TC, and SK). Thus, they used their experiences as a research resource, but remained cautious to prioritize the participants’ narratives. 

### 2.4. Recruitment of Participants

A total of 83 participants were recruited from March to May 2020 through professional and social networks. The criteria for inclusion were (i) self-identifying adult Chinese immigrants, which included all persons identifying as having Chinese ancestry and not living in their country of ethnic origin; (ii) residency in the GTA; and (iii) willingness to participate in online interviews in their preferred language. 

Note that the researchers did not anticipate it would be necessary to analyze all 83 interviews from the original study, as we applied a different paradigm (i.e., critical realism) to answer a narrower research question here as compared to the original qualitative study. Hence, 35 of the 83 interviews were estimated sufficient to achieve theoretical adequacy (with maximum variation in age, gender, country of origin, occupation, years residing in the GTA, and those reporting direct or indirect experiences of racial discrimination). In fact, thematic saturation was achieved at 36 interviews. Following Vasileiou et al. [23], theoretical saturation was determined when three of the research team members (DL, SK, and HH) reached consensus on conceptual adequacy. This was decided through the triangulation of intrinsic methodological sources, including maximum diversity of data, richness and volume of data, and thematic saturation (when no ‘new’ theoretical patterns emerged) after the analysis of 36 interviews [23]. 

### 2.5. Data Collection

Prior to data collection, ethical approval was granted by the respective Research Ethics Boards of the participating universities. Interview data from the original 83 participants were collected by phone, Zoom, Google Meet, or Skype (audio only) in English (64 interviews), Mandarin (5 interviews), or Cantonese (14 interviews). The research interviewers collected the socio–demographics followed by interviews (on average 45 min each). The semi-structured interview questions concerned constructs of risk perception, social determinants, cultural racism, and stigma, using prompts to gather in-depth information following the participant’s lead. The audio recordings were transcribed verbatim and—when necessary—translated into English and verified by two researchers fluent in Chinese and English. Data management and organization were supported by NVivo12 software [24]. 

### 2.6. Data Analysis

Data analysis followed an iterative process through four phases following Fryer [25]: (i) familiarization with the data; (ii) apply, develop, and review codes; (iii) develop and review themes; and (iv) generate conclusion and reports. Briefly, the lead author led the familiarization with the data with at least one other researcher, by skimming all the data and documenting initial questions, insights, associations, and surprising constructs. 

The second level of analysis generated codes for chunks of text. The initial coding was applied to 10 percent of the original 83 interviews (nine of them) for preliminary descriptive constructs [25]. These nine interviews were part of the chosen 35 interviews (to maximize variation in the theoretical constructs, as previously stated). These interviews were initially coded independently, and then we discussed codes for comparison and to reach consensus (the layer of the experiential or ‘empirical’). These codes laid the groundwork for the initial coding structure. 

The third level of analysis comprised an iterative process of cycling back and forth between familiarization, developing codes, and developing themes. We grouped similar codes together in what Fryer [25] calls the ‘standardization’ of codes, whilst ‘consolidation’ meant that we questioned how the codes might be used to address the research question at the level of the ‘actual’ [25]. 

The fourth level required that we use retrodictive (deductive) reasoning to confirm the theoretical constructs. Our conclusions were drawn from previous research on risk communication and the related literature, to consider the explanatory power of our posited themes to generate material or (in)tangible events (the layer of ‘the actual’). Our reports reflect the multiple mechanisms and structures of our theorized model (layer of ‘the real’) [25]. 

Note that today’s Chinese diaspora may have a tendency to maintain their identification with their ancestral homeland, as well as with other multiple homelands, even after many years of living outside their country of origin, regardless of whether they were born in Canada or spent many years outside their country of origin [26]. Hence, data analysis to parse out the differences between identification with their Chinese ancestry and immigration status was not conducted. 

### 2.7. Trustworthiness

We adhered to strategies to ensure four criteria were met, following Fryer [25]: (i) credibility; (ii) plausibility; (iii) transferability; and (iv) utility or impact. The first two criteria are commiserate with the validity and reliability of our coding processes. This entailed that we conducted independent and comparative coding to reach confirmation of all our codes for the first ten percent of transcripts. This laid the foundation for our descriptive coding thereafter. Further, the plausibility of the theoretical patterns was ensured through active discussions between at least two members for all the interviews, whereby we conducted theoretical memoing of our reflexivity and insight as codes related to ‘new’ knowledge and its relationship to the related health communication literature. Several rounds of peer debriefing expanded the theoretical constructs within the broader context of risk literacy literature and storytelling interventions addressing illness and/or underserved communities. Finally, to promote transferability, we present, in this manuscript, the raw participant data and a brief description of the participants’ contexts. The utility or impact was assessed through analysis of how the findings agreed or disagreed with and extended the knowledge of the phenomena, thereby reconceptualizing the main theoretical construct. In what follows, we adhered to the EQUATOR Standards of Reporting Qualitative Research devised by O’Brien et al. [27] to ensure transparency and comprehensiveness. 

### 2.8. Ethical Aspects

This study was approved by the respective Research Ethics Review Boards of the participating universities. Data collection was proceeded by obtaining written informed consent from all participants. Confidentiality was ensured through the storage of data on password-protected servers and computers. Further, data were shared on secure password-protected internet platforms. Last, participants were referred to only by their interview number, sex, and their experiences of stigma or racism, the factors shaping their magnitude of cognitive dissonance.

## 3. Results

### 3.1. Participants’ Socio–Demographic Characteristics

The findings arere based on 36 participant interviews. Sixty-four percent of the participants were aged between 25 and 64 years. Sixty-four percent were female. Forty-two percent had immigrated from Hong Kong, thirty-one percent from mainland China, and eight percent from Taiwan. Fifty-three percent were employed. Seventy-five percent had lived in the GTA for six years or more. Finally, seventy percent reported having experienced direct or indirect stigma, racism, or microaggression. 

**Figure 1:**
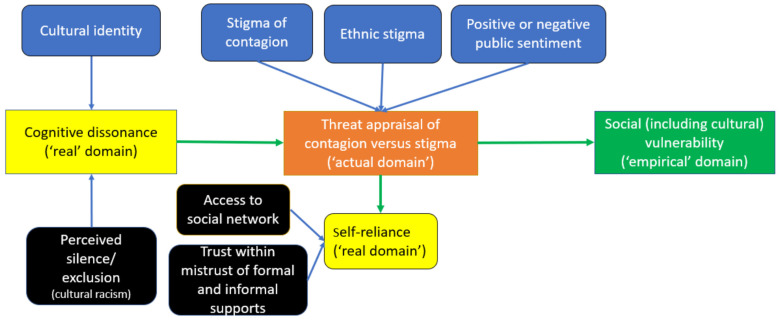
Theoretical model of Chinese diaspora persons’ risk perception and threat appraisal generating their social vulnerability during the first wave of COVID-19 [14].

As stated previously, part one of our study [14] revealed that all participants sought and appraised information with some degree of cognitive dissonance regarding both COVID-19 contagion and cultural racism. Their cognitive dissonance moderated their experiences of social (including cultural) vulnerability. (Please see Figure 1 for details of part one of our study’s theoretical findings.) 

Those who described a greater degree of cognitive dissonance appeared to exhibit heightened risk perceptions of public stigma (disapproval of or discrimination against oneself by others) and/or self-stigma (disapproval of or discrimination against oneself as a group member) than that of their risk appraisal of COVID-19. 

Herein, we explain how the participants’ different cognitive dissonance pathways shaped their risk perceptions to generate their social (including cultural) vulnerability. Specifically, we expand on the key components that shaped the participants’ threat appraisals and self-reliance, leading to their social vulnerability. These were (i) their cultural identity recognition and (ii) (mis)trust in authorities’ information. 

### 3.2. Cultural Identity Recognition

Cognitive dissonance was problematic to the degree that the participants perceived their actions as contrary to societal norms during the first wave of COVID-19. These participants closely identified with an Asian ethnic identity and familial duty to wear a protective mask in public. This manifested in the participants’ anxiety, fuelling their reluctance to venture outside their homes and indecision regarding whether they should wear a mask, following social media reports of cultural racism against Asian persons:

*‘I believe I heard a news report [CNN World News] that somebody from London [UK] got attacked, punched by a crazy person… Just because he was Asian and wearing a mask… By that time, the outbreak wasn’t in Canada or wasn’t in Toronto yet so I was thinking, well, maybe it’s not that mandatory [that] I’ll have to wear the mask. Maybe for self-protection, I put a mask in my pocket just in case… if somebody’s coughing or looks sick, and yeah, but I can’t. I was too scared to put on my mask. Too scared.’*

(#39, female student, resident of GTA for 5 years)

Contrasting cases were revealed when participants remarked that they felt a degree of separation from their Chinese communities due to not identifying with certain cultural aspects, such as the Chinese dialect or traditional beliefs. For instance, one participant remarked

*‘I’m seeing it through the CBC [Canadian-born Chinese] lens…Because I’m not tight with that, you know… you go down to the market and down in Chinatown to [name of store]. And no line up to get in but people are also not regulating the same kind of awareness of physical distancing, they were wearing gloves, they were doing things, but the aisles are so narrow they don’t have that sort of Western medical sensibility. And so, we actually felt kind of scared going in there. We were holding our breath more.’*

(#45, male, resident for 32 years)

As the above excerpt states, the environment’s social cues aroused the participants’ fears, including non-verbal stares and inattention to physical distancing in public. These environmental cues were moderating factors contributing to participants’ anxiety and cognitive dissonance. 

In a contrasting case, participant #46 (below) argued that public stigma was related not to ethnicity but rather to the public trust granted to cultural identification with one’s socio–economic class and neighbourhood.

*‘I think it is not racial. It is because of the class… Maybe if I was taking the bus in China town, the drivers wouldn’t really be so polite. My area is mostly WASP [White Anglo-Saxon Protestant], they are very polite, even if I am Asian-looking.’*

(#46, female, resident for 54 years)

### 3.3. (Mis)trust in Authorities’ Information

Individuals expressed fear and mistrust regarding the information conveyed to them about COVID-19, which impacted their sense of responsibility for seeking public help when needed, instead seeking information from their informal social network to appraise information accuracy. One participant, who had immigrated to the GTA fewer than three years previously, expressed difficulty in trusting ‘official news’ when it contradicted their prior experiences and beliefs formed during the SARS outbreak:

*‘Actually, put it this way, I always look at the official news first, no matter what it is. There are some facts, and I would look for more on my own. I think Canada is neutral up until now [May 2020]. I read the government news there, even Dr. Teresa Tam said not to wear masks, the government promoted that don’t wear mask if you are not sick, this…because I came from Hong Kong, I experienced SARS. When it seriously contradicts my opinions, I tend not to believe it, or I would look at and check with the media that I can trust.’*

(#66, female, resident for 2.5 years)

The duration of the participants’ residency in Canada did not necessarily alter their mistrust of public health authorities, as one participant, who had lived in the GTA for over 20 years, stated

*‘I would say in terms of the channels, news, mostly I hear them, but, like, do I 100% trust it? I don’t. I have personal opinions.’*

(#14, female, resident for 22 years)

As the above excerpts suggest, many participants felt conflicted about trusting news from public health authorities. Overall, they expressed mistrust in the authorities, who were perceived as evading or avoiding detailed public discourse (e.g., statistical, contextual) regarding COVID-19. Most participants evaluated their opinions based on their informal reference groups, such as family, friends, or colleagues. Below, we explain how individuals’ cognitive dissonance contributed to diverging self-reliant responsibilities to protect themselves and their significant others from potential harm from contagion and/or cultural stigma. 

### 3.4. Diverging Pathways/Types of Cognitive Dissonance

Our findings reveal three different emotional responses to appraising the threat of contagion versus public stigma. *First*, fear, shame, and dissociation from their cognitive dissonance of anticipatory stigma elicited hesitancy and isolation from their cultural communities. This response generated uncertainty or avoidance regarding their responsibility (or not) to act against the potential harms of the COVID-19 contagion and/or public stigma. *Second*, the rationalization of and ambivalence toward their cognitive dissonance of public stigma led some to maintain a kind of status quo. This was characterized by accepting some degree of public stigma as rational and ‘normal’ and, thus, ‘not necessarily my responsibility.’ *Third*, the externalization of their cognitive dissonance generated resistance to and distancing from their internal conflicts. This created possibilities for deconstructing and reauthoring their vulnerability; thus, their responsibility was to counteract and critically question taken-for-granted social norms that were perceived as harming them as individuals who identified with their ethnic communities.

1. Fear, shame, and dissociation from cognitive dissonance generates hesitancy and isolation.

For some participants, fear mediated whether and how they engaged with the wider public’s expectations of them as Chinese and their responsibilities as citizens:

*‘Of course, for the sake of my health and other people’s health, I should follow the government’s instruction, for example, keep social distance. But my opinion is that, given so many racists going against Chinese because of COVID-19, I need to behave in the best way so that those racists have no excuses to find fault in me.’*

(#38, female, resident for 30 years)

Another participant admitted that although he had ‘not yet’ experienced stigma, staying at home appeared to be in his favour:

*‘It hasn’t happened yet. I haven’t experienced anything like that. But one thing is because I stay at home almost all the time, so I don’t have any interaction with outside people, so that’s part of the reason.’*

(#56, male, resident for 50 years)

As the excerpts illustrate, the participants were conflicted between their responsibility to remain safe from the COVID-19 contagion and their responsibility for their physical safety in anticipation of cultural racism. This created ambivalence as to how they should behave. 

Some participants appeared to internalize cultural racism through shame or dissociation from Asian communities similar to theirs. This assigned the responsibility for controlling public stigma to oneself and one’s significant others. One participant described the internalized racism in her family, who isolated themselves from communities similar to their own:

*‘I do see like—just sometimes even internalised racism against Mainland Chinese people. I think a good example would be when the whole COVID stuff started, like my family sending a chat, my extended family sending a chat being like “We should avoid places where Chinese people hang out”.’*

(#18, female, resident for 1.5 years)

As the excerpt suggests, isolating or dissociating oneself from one’s cultural community or the spaces in which they gather was aligned with the responsibility to protect oneself against the COVID-19 contagion and public stigma.

2. Rationalization of and ambivalence (uncertainty) toward cognitive dissonance maintains status quo.

The degree to which participants experienced cognitive dissonance appeared to be based on their sense of responsibility for anticipating stigma when appraising information. If they were unable to do so, they rationalized that it was not their responsibility to do so. This allowed them to maintain a degree of the ‘status quo’ by accepting some degree of public stigma as ‘normal.’ They thus remained in their ‘comfort zone’ and were absolved from the responsibility to be concerned about it. For example, participant #17 stated

*‘When I went to the supermarket, people stayed far from us early in March when we wore masks. But yes, they don’t look nice, but I don’t—I don’t think that’s a bad thing either. Because they’re trying to protect themselves.’*

(#17, male, resident of the GTA for 28 years)

As participant #17 observed, he accepted a certain degree of tolerance for public stigma. By contrast, another participant (below) rationalized that some Asian immigrants did not ‘take an interest’ in mainstream beliefs and were thus indifferent to public stigma:

*‘I guess it depends, because some of the immigrants…don’t come into contact with a lot of foreign—like, the mainstream society. Maybe they run a restaurant business, you will have their own food, they are all Chinese. Right? …They are not interested in changing or they are not interested to say protest or anything like that. They are not interested. They are very comfortable in terms of staying in their own small circle.’*

(#27, male, resident for 7 years)

In these instances, a degree of cognitive dissonance appeared acceptable, perhaps through socialized tolerance based on the notion that some measure of public stigma was ‘normal’.

Similarly, the avoidance of stigma relegated the responsibility for their cognitive dissonance to others, allowing oneself to be absolved from engaging with the tensions driven by it. For example, participant #66 stated

*‘Frankly, I don’t think more people standing out would make a difference [to stigma]. I would think, what is next after you say it? It will only result in useless arguments.’*

(#66, female, resident for 2.5 years)

In another case, a participant and member of the clergy rationalized the responsibility for avoiding confrontation as part of the cultural norm of ‘being Chinese’,

*‘We [Chinese] might have a culture that is easy going and it is not easy for a Chinese to go to a place and complain or report or go to the government to complain. We do not have that.’*

(#28, male, resident for 25 years)

As the excerpt suggest, the cultural norms associated with ‘being Chinese’ could deter or absolve one from taking responsibility for an outcome perceived as outside of one’s control. 

By contrast, the ambivalence or uncertainty about responsibility for public racism was rationalized by one participant as ‘not worth caring about’ until its injurious effects were made visible to others like themselves. As participant #14 expressed

*‘I don’t think we really care about the stigma unless we get bullied.’*

(#14, female, resident for 22 years)

As the excerpts reveal, one’s responsibility to act on their cognitive dissonance depends on the urgency and degree of consequential harm, and when and if one’s responsibility for their cognitive dissonance could be left unchanged (i.e., status quo).

3. Externalizing cognitive dissonance through resistance and distancing facilitates deconstructing and reauthoring (reassigning) responsibility.

Resistance to stigma was demonstrated by a refusal to adopt stereotypes of cultural racism, perhaps due to collective traumatic memories from past pandemics. For example, participant #62 stated

*‘As soon as he saw me, he covered his face and then ran away. So, once I saw that, I was like-…I was really shocked because Toronto, right?… This is the biggest city in Canada and then the most multicultural city in Canada—in the world, I would say. I’m surprised that people react like that. I’m shocked more than disappointed.’*

(#62, female, resident for 7 years)

As the excerpt suggests, some participants drew on their national identity and the values of diversity and inclusivity when evaluating and resisting public stigma; thus, they reauthored their responsibility connected to their citizenship rights and advocated against it. 

Similarly, externalizing efforts to distance oneself from the internal conflicts caused by public stigma were humorously described. To make light of the stigma was to question its credibility and emotionally distance oneself from it. This allowed individuals to emotionally coping with their fears and reframe public stigma as separate from their self. As such, one could reassign ambiguity about responsibility for it (reauthor) to others. One male participant recounted how he counselled his son, who had experienced stigma on public transit:

*‘I thought it [overt stigma] was really funny, but they were being jerks. But it was really funny because they would like oh they wanted a seat, they’d start coughing and people would form of black plague ring around them like, whoa, empty seat and they sit down and they start laughing to themselves because they knew people are freaking out. Like, hey, you’re going to look at me because I’m Asian and I’m coughing. And you’re going to treat me like that? I’m going to take your seat.’*

(#45, male to son, the former a resident for 32 years)

When the son was enabled to distance himself from the public racism, he discerned that he was *not* obliged to take responsibility for it. 

Relatedly, some participants advocated inclusivity not only for themselves but to generally combat discrimination against Asian communities in their places of residence or in schools:

*‘Well, I don’t think the Chinese community needs to be treated like any differently…But I don’t think you should do anything differently the way you treat Chinese people. I think maybe just like, I don’t know how to destigmatise it, but yeah, like more awareness.’*

(#51, female student, resident for 11 years who denied racism)

*‘I think it should be done at some very grassroot events… It could affect the questions in Liberal Education, we could teach the children we shouldn’t discriminate, and then we have these different cases. If they have gone through this process, it would be easier for them.’*

(#66, female, resident for 2.5 years)

As the excerpts reflect, some participants had a strong sense of duty to advocate for equity among all communities, irrespective of ethnicity or age. One participant (#80) recommended storytelling to raise awareness of and empathy towards Asian communities:

*‘So together with these so-called exchanges and advocacy, I think this is a really hard thing to do. I personally like this to take place in the form of a video or use a video to tell a story. It is more straightforward and figurative.’*

(#80, female student, resident for less than one year)

Below, we shall explore how the findings may be theoretically aligned with storytelling (narrative) interventions, as a ‘culturally safe’ way to illuminate cognitive dissonance; we argue that revealing hypocrisy in one’s beliefs and actions (i.e., manifestation of cognitive dissonance) may alter attitudes and behaviours, in both oneself and others, through the mediation of responsibility. 

## 4. Discussion

Our findings extend the theorization of cognitive dissonance arousal (direct and indirect) that occurred during the first wave of COVID-19 for the Chinese diaspora in Canada’s GTA. Specifically, our findings emphasize participants’ hypocrisy in trusting, yet mistrusting, public health information. Given that trust is vital to engaging with public dialogue about risk messaging [28], we posit that a vicarious identification with people of Asian ancestry could magnify the participants’ propensity to mistrust government-authorized information. Indeed, Cooper [15] suggests that cognitive dissonance can be vicariously generated in line with social identity theory. Individuals who consider themselves part of a group tend to share common values and identify with the group’s actions to control for anticipated consequences [15]. This response is affirmed by the EPPM model [8]; the rejection of the message is defensively motivated, due to fear dominating protective motivations. Hence, public stigma surrounding cultural identification (e.g., ethnicity, socio–economic status, and neighbourhood) contributed to the participants’ cognitive dissonance. 

In line with Cooper [15], we posit that the greater the participants’ arousal, the greater their need to ease their cognitive dissonance. In particular, we posit that the magnitude of the participants’ felt cognitive dissonance was mediated by their perceived responsibility (part of self-efficacy) and whether they anticipated being able to deflect public stigma from themselves (response-efficacy). Those who reported fear, shame, and dissociation were hesitant or chose to isolate themselves from the discourse that perpetuated Asian public stigma, which seemed beyond their control. Meanwhile, those who experienced cognitive dissonance to a lesser degree rationalized and abdicated their responsibility for engaging with public stigma. As such, they did not appear to experience a strong personal sense of duty to alter their attitudes or behaviours in response to public stigma. Finally, participants’ resistance against internal cognitive dissonance appeared to arouse in them a new meaning or purpose, a sense of duty to advocate for equity focused on the Chinese community, not only for themselves, but for a broader social network based on their place of residence and educational background. By mapping our results onto the theoretical components of cognitive dissonance [28], we explore storytelling interventions (suggested by one of our participants) to potentially transform and/or mitigate cognitive dissonance.

### 4.1. Mapping Our Results to Risk Perception Targeting Cognitive Dissonance

According to Wood and Miller’s [28] review of disaster communication, there are five factors for risk perception that are actionable in regard to cognitive dissonance: (i) identity recognition, (ii) reasonable fear, (iii) audience participation, (iv) accuracy motivation, and (v) storytelling. 

Identity recognition concerns the targeted community’s cultural and ethnic composition and how they process the facts communicated by authorities [28]. Fear, within reason, can support preparation for and discussion of risk communication; however, if the risk is not considered imminent, cognitive dissonance can neutralize the responsibility to act [28]. As our study demonstrates, some participants refrained from taking precautions against COVID-19 (e.g., wearing masks) when they experienced cognitive dissonance (as fear, shame) regarding the increased risk of public stigma. When fear turned to self-stigma or dissociation from their Asian communities, cognitive dissonance appeared to hinder further engagement with risk communication (i.e., hesitancy or ambivalence). 

Cognitive dissonance can only be reduced by audience participation when it enhances self-efficacy to adopt public health measures and actively participate in finding solutions [28]. In our study, the participants’ resistance and distancing opened up possibilities for new meaning, including advocacy, aligned with taking protective action or preventative behaviours against the COVID-19 contagion for themselves and their families (e.g., advocating for equity and understanding of Chinese communities). 

Accuracy motivation refers to an audience’s confidence in the credibility of authoritative risk communication [28]. The need for consistent information during the initial risk communication phase is crucial for whether the audience will seek alternative information sources [28]. All the participants in our study expressed some mistrust of formal risk communication and had sought alternative information sources on which to base their perceptions of risk and the need to take protective action. 

In storytelling, the audience enacts scenarios to support several substantiated possibilities to which they can relate and for which they can take ownership/responsibility to prepare themselves for risks [28]. On the whole, our findings reflect the narration of fears aroused by the struggle to comply with public health guidance due to the limited concrete, effective, and feasible ways to do so when barriers were encountered, such as the potential stigma around mask wearing. We posit that these experiences signified their cognitive dissonance and divided their families and friends into groups who magnified or denied the potential harms of COVID-19. As Woods and Miller [28] stated

*‘When reality clashes with our deepest convictions, we’d rather recalibrate reality than amend our worldview. Not only that, but we become even more rigid in our beliefs than before’*

(p. 44).

### 4.2. Implications for Health Policy

By mapping our study results onto Wood and Miller’s [28] risk perception factors, we suggest a potential application for training moderators to be ‘good’ storytellers, so that when hearing a story that resonates with oneself, it may alter one’s cognitive dissonance and thereby one’s social vulnerability. Indeed, storytelling is an interpersonal experience; ‘for in truth, we tend to hear another’s story with our own stories, our lenses, as it were, shaping and refining the content and tone of what we are encountering when we hear the story of another’ [29] (p. 535). Thus, we were directed to explore storytelling (rooted in the narrative therapy of White and Epston [30]) as an intervention with which to mitigate cognitive dissonance.

According to Wood and Miller [27], ‘the story behind the message is as important as the content of the message itself’ (p. 47). In our study, the narratives surrounding cognitive dissonance generated fear, which was not in itself necessarily pernicious in all cases. Cognitive dissonance has consistently been demonstrated as beneficial for individuals wishing to minimize the stigma surrounding their weight or to modify their smoking habit [9,31]. Thus, while cognitive dissonance evokes a certain degree of ‘healthy’ fear, an excess is overwhelming, masks the danger, and provokes denial [28].

Cognitive dissonance may also be correlated with the duration of perceived risk, which, in the case of COVID-19, was prolonged and theorized as potentially de-intensifying our perceived responsibility to respond accordingly [28]. However, pre-existing propensities towards cultural cognitions rooted in identification with a social group are salient, and may continue to alter attitudes and behaviours in response to the evolving situation [29]. 

Storytelling’s power lies in the risk communicator’s capacity not only to elicit fear of anticipated negative outcomes, but to strategically direct the audience towards ways of developing empathy for oneself and others, and externalizing responsibility through resisting or distancing oneself from one’s vulnerability through alternative choices and actions (as demonstrated by some of our participants who used humour to distance themselves from the threat of public stigma). According to Nunn [32], transformative potential occurs when one understands the need for assumptions about the other, even if one does not necessarily endorse them for oneself. Indeed, self-disclosure of past hypocrisy provides the background of cultural humility, foregrounding the individual’s trustworthiness as a role model. Hence, storytelling regarding one’s social vulnerability can foster exchanges that reinforce activism when one identifies with the role model and can reduce cognitive dissonance for oneself; in doing so, it creates new expectations/norms [33].

We advocate that training by moderators is needed to create an environment that safely targets cognitive dissonance, allowing for cultural differences to emerge in a ‘timely’ way. For instance, unlike ethnic jokes used to establish dominance and reinforce race conflict, self-deprecating ethnic humour in intra- or inter-ethnic relations can simultaneously take ownership of and challenge and transcend prevalent racist discourses [34]. Esholdt [34] states that a humorous framing of stereotypes reduces tension and allows one to transcend the very stereotypes that serve to marginalize them. Consequently, ‘humour can defuse ethnicity as a sensitive subject’ [34] (p. 697). While self-directed humour is not novel, we suggest that it constitutes an important means of implicitly discussing cultural differences in a culturally safe manner, though advanced preparation and skill are required to ensure appropriate ‘timing’. 

Like humour, some may use storytelling as an effective form of interpretive labour, potentially acting on persons vicariously to better their attitudes and behaviours; for others, however, storytelling may be offensive or harmful. Indeed, we argue that care should be taken to train select peer leaders who are both morally and scientifically credible within their communities to serve as role models and mentors for storytelling with respect to cognitive dissonance. Doing so may prevent interpretive power from further marginalizing those who do not fit within the shared narrative, as the stories become the shared norm rather than idiosyncratic [35]. Further, while some may find emotional catharsis beneficial, it may induce others to provide unfavourable feedback or behave rudely, and even reinforce disinformation [36]. Moreover, while stories may confer dignity to the tellers, it may also uncritically valorize the individual story and inadvertently negate the complex relationship between narrative and truth [37]. Thus, moderators should not be culturally confused as to their identity and should be emotionally ready to tell their stories [38] and evoke a responsibility to help individuals come to terms with their cognitive dissonance and transform the tensions in their lives.

## 5. Conclusions

As shown in the literature, our study confirms that cognitive dissonance can be reduced when individuals compare their behaviour to the personal and normative standards of the larger (national) society with which they identify and in which they reside [39]. Perceived travel restrictions during COVID-19 were considered a norm for the Chinese diaspora in the Netherlands, and they adjusted their attitude positively to preserve their trust in the host country and resolve their cognitive dissonance [40]. Accordingly, we suggest that the achievement of sustainable ‘new’ social norms to mitigate COVID-19 requires evoking the dissonance-based motivational drive of hypocrisy, as one of our study participants demonstrated with humour (satire). Thus, narratives can vicariously trigger cognitive dissonance, generating the responsibility to act on the moral prosocial behaviours of society that mitigate adversity resulting from social and cultural vulnerability. 

We suggest that sharing stories that capture one’s dissonance-based motivational drive can facilitate learning how to externalize and deconstruct one’s cognitive dissonance. Indeed, the mechanisms of cognitive dissonance can modify risk perception and mitigate social and cultural vulnerability, thereby averting potential long-term negative consequences for one’s mental health and well-being. We hope our guidance, to train educators to target pathways of cognitive dissonance and draw on storytelling (with humour), can assist them to better convey information in ways that are more inclusive during public health emergencies. 

## Data Availability

Data supporting the findings are available within the article [and/or] its associated materials.
